# Characteristics and Outcomes of 35 Breast Cancer Patients Infected With COVID-19

**DOI:** 10.3389/fonc.2020.570130

**Published:** 2020-10-21

**Authors:** Bo Zhang, Rong Xie, Shawna M. Hubert, Yuanhang Yu, Yue Zhang, Xiao Lei, Wei Deng, Jianying Chen, Yunqiao Li

**Affiliations:** ^1^Department of Breast and Thyroid Surgery, Tongji Medical College, Union Hospital, Huazhong University of Science and Technology, Wuhan, China; ^2^Department of Thoracic Medical Oncology, Department of Genomic Medicine, MD Anderson Cancer Center, The University of Texas, Houston, TX, United States; ^3^Department of General Medicine, Tongji Medical College, Union Hospital, Huazhong University of Science and Technology, Wuhan, China; ^4^Department of Gastrointestinal Surgery, Tongji Medical College, Union Hospital, Huazhong University of Science and Technology, Wuhan, China; ^5^Department of Geriatrics, Tongji Medical College, Union Hospital, Huazhong University of Science and Technology, Wuhan, China

**Keywords:** SARS-CoV-2, non-cancer, breast cancer, clinical characteristics, prognosis

## Abstract

Since December 2019, a novel coronavirus disease (COVID-19) caused by severe acute respiratory syndrome coronavirus 2 (SARS-CoV-2) has rapidly engulfed the world. Cancer patients infected with COVID-19 are considered to carry higher severity of the disease and higher mortality rate than common COVID-19 patients in previous studies. However, due to the poor clinical information on COVID-19 patients with cancer, the evidences that supported this conclusion are insufficient. At present, rather limited reports have analyzed the clinical data of breast cancer patients infected with COVID-19. Therefore, in this retrospective study, we described the clinical characteristics and the outcomes of 35 COVID-19 patients with breast cancer and compared 55 COVID-19 patients without cancer and 81 COVID-19 patients with other types of cancer as controls. Our data showed that there were no differences in disease severity and outcomes between the COVID-19 patients with breast cancer and the common COVID-19 patients, which was in contrast to previous studies. In addition, compared with other types of cancer patients, asymptomatic infections and mild cases among breast cancer patients made up a substantially larger proportion. Our results indicated that the clinical characteristics of breast cancer patients were milder than those of other types of cancer patients, but there were no significant differences in outcomes between the two groups.

## Introduction

In December 2019, a novel coronavirus disease (COVID-19) that originated from severe acute respiratory syndrome coronavirus 2 (SARS-CoV-2) infection was first reported in Wuhan, Hubei province in China. The World Health Organization (WHO) announced the outbreak of COVID-19 as a public health emergency of international concern on January 30, 2020 (1). By May 18, 2020, the rapid spread of SARS-CoV-2 worldwide had resulted in more than 4.6 million confirmed cases and more than 312,000 deaths in 216 countries and areas (2, 3). The clinical manifestations of COVID-19 are varied, including fever, cough, sore throat, fatigue, diarrhea, myalgia, and dyspnea (4). However, many COVID-19-infected patients were asymptomatic; previous studies indicated that asymptomatic infections account for about 17.9–30.8% of all cases (5).

During this COVID-19 epidemic, patients with cancer were more vulnerable to the harm by COVID-19 infection. A mount of reports had demonstrated that cancer patients were at a higher risk of COVID-19 infection compared with persons without cancer (6, 7). Furthermore, oncologic patients were more likely to carry high severity of the disease and poor prognosis in case of immunocompromise as a result of anti-tumor therapy such as chemotherapy and radiotherapy (8). However, it must be emphasized that data on COVID-19-infected patients with cancer were poor; thus, the evidence for this conclusion was insufficient (8, 9). Kalinsky et al. reported 27 cases of breast cancer patients with COVID-19, in which 20 cases (74%) did not require hospitalization and 26 cases (96.3%) had a good prognosis (10). To date, few researchers described the clinical characteristics and the prognosis of breast cancer patients with COVID-19 or compared with non-cancer patients or with other types of cancer patients. Due to the rather limited size of samples and clinical information, there were still some questions that remained unclear and needed to be answered, such as whether these patients had different clinical processes and outcomes.

Therefore, in this retrospective study, we described the demographics, clinical features, treatment, and outcomes of 35 breast cancer patients with COVID-19. In addition, we sought to explore the differences in clinical characteristics and prognosis between COVID-19-infected breast cancer patients and other types of cancer patients and non-cancer patients. This study may help clinicians better understand the impact of COVID-19 on breast cancer patients and lead to a more efficient distribution of medical resources.

## Methods

### Methodology and Participants

Retrospective study is a research method that takes the present as the result and traces back to the past. Our study was performed in five designated tertiary hospitals for the treatment of COVID-19 in Wuhan, China. Thirty-five breast cancer patients with COVID-19, 81 other types of cancer patients with COVID-19, and 55 COVID-19 patients without cancer were recruited between January 17, 2020 and May 18, 2020. All the enrolled patients were laboratory-confirmed COVID-19-infected cases as they were positive in nucleic acid testing or antibody test for SARS-CoV-2. Nasal and/or pharyngeal swab specimens were collected and tested for SARS-CoV-2 by real-time reverse-transcription polymerase-chain-reaction assay. The diagnoses of COVID-19 infection and of patients' clinical condition were based on the updated New Coronavirus Pneumonia Prevention and Control Program (trial version 7). This study was approved by the Ethics Committee of Tongji Medical College of Huazhong University of Science and Technology.

### Data Collection and Definitions

Clinical data including demographic features, clinical characteristics, laboratory examinations, chest CT images, treatment, and outcomes were obtained from medical records. Laboratory examinations were performed within 24 h after admission. The clinical outcomes of these patients were monitored until May 18, 2020, the final follow-up date for all patients to be discharged. All data were reviewed and verified by two physicians independently. Patients with asymptomatic SARS-COV-2 infection were defined as those positive in nucleic acid testing or antibody test for SARS-CoV-2 but without clinical symptoms of pneumonia or CT imaging pattern such as patchy shadow and ground-glass opacity.

### Data Analysis

Continuous variables were presented as mean with standard deviation or median with interquartile range (IQR); categorical variables were presented as counts and percentages. When the data were normally distributed, independent group *t*-test was used to compare continuous variables; otherwise, Wilcoxon rank-sum test was used. Chi-square test or Fisher's exact test (due to the small size of the samples) was applied to categorical variables as appropriate. All statistical analyses were done with SPSS Statistics, version 23.0. A two-sided *p*-value <0.05 was considered statistically significant.

## Results

### Demographic and Baseline Characteristics

A total of 35 COVID-19 patients with breast cancer were enrolled in this study. The demographic and baseline characteristics are shown in Table 1. The median age was 56 years (IQR, 42–62); all of them were female. All patients were from Hubei province, China. Among them, 30 cases (85.7%) were living in the three main districts of Wuhan: 21 (60%) in Hankou, six (17.1%) in Hanyang, and three (8.6%) in Wuchang. Within 1 month of COVID-19 diagnosis, six (17.1%), two (5.7%), and two (5.7%) received chemotherapy, radiotherapy, and targeted therapy, respectively. More than a third of the patients (13, 37.1%) had comorbidities, including hypertension (six, 17.1%), myelosuppression (four, 11.4%), diabetes (four, 11.4%), anemia (three, 8.6%), cardiovascular disease (two, 5.7%), cerebrovascular diseases (one, 2.9%), liver dysfunction (one, 2.9%), and chronic bronchitis (one, 2.9%).

**Table 1:** Demographics and baseline characteristics of 35 breast cancer patients with COVID-19.

**Characteristics**	**Value**
**Age**	
Median (interquartile range)—year	56 (42–62)
**Sex**	
Female	35 (100%)
**Residential area**	
Wuhan	
Hankou	21 (60%)
Wuchang	3 (8.6%)
Hanyang	6 (17.1%)
Outside Wuhan	5 (14.3%)
Chemotherapy within 1 month	6 (17.1%)
Radiotherapy within 1 month	2 (5.7%)
Targeted therapy 1 month	2 (5.7%)
**Comorbidities**	
Hypertension	6 (17.1%)
Myelosuppression	4 (11.4%)
Diabetes	4 (11.4%)
Anemia	3 (8.6%)
Cardiovascular disease	2 (5.7%)
Cerebrovascular diseases	1 (2.9%)
Liver dysfunction	1 (2.9%)
Chronic bronchitis	1 (2.9%)
Asymptomatic	24 (68.6%)
Clinical manifestations of COVID-19	11 (31.4%)
Fever	6/11 (54.5%)
Cough	8/11 (72.7%)
Fatigue	6/11 (54.5%)
Chest tightness	4/11 (36.4%)
Myalgia	3/11 (27.3%)
Diarrhea	3/11 (27.3%)
Chills	2/11 (18.2%)
Shortness of breath	2/11 (18.2%)
Dyspnea	2/11 (18.2%)
Anorexia	1/11 (9.1%)
Headache	1/11 (9.1%)
Hemoptysis	1/11 (9.1%)
**Disease severity**	
Mild	24 (68.6%)
Severe/critical	11 (31.4%)

As shown in Table 1, most patients (24, 68.6%) were asymptomatic at the onset of COVID-19. Among the remaining 11 patients with clinical symptoms, six (54.5%) presented with fever, eight (72.7%) presented with cough, six (54.5%) presented with fatigue, four (36.4%) presented with chest tightness, three (27.3%) presented with myalgia, three (27.3%) presented with diarrhea, two (18.2%) presented with chills, two (18.2%) presented with shortness of breath, two (18.2%) presented with dyspnea, one (9.1%) presented with anorexia, one (9.1%) presented with headache, and one (9.1%) presented with hemoptysis. Among 35 breast cancer patients with COVID-19, 24 (68.6%) were mild and 11 (31.4%) were severe/critical.

### Laboratory and Radiologic Findings

The blood routine results showed lymphopenia in 10 patients (52.6%) and thrombocytosis in two patients (10.5%). Decreased levels of white blood cell count were observed in two (10.5%) and elevated levels of that were observed in two (10.5%). Monocyte count was suppressed in one (5.3%) and elevated in three (15.8%). Three cases (15.8%) showed increased levels of neutrophil count, and two (10.5%) had decreased levels. In terms of data on blood biochemistry, we found that alanine aminotransferase (ALT) was shown to be increased in five patients (26.3%), and aspartate aminotransferase (AST) was shown to be increased in three (15.8%). Creatine kinase (CK) was increased in two (10.5%), lactate dehydrogenase (LDH) was increased in six patients (31.6%), and both CKMB and cardiac troponin (TNI) were within the normal range. The infection biomarkers of C-reactive protein (CRP) were elevated in five (50%), and procalcitonin was increased in two (28.6%). Decreased levels of CD3^+^ T cells, CD4^+^ T cells, and CD8^+^ T cells were observed in two (22.2%), one (11.1%), and two (22.2%), respectively. Other abnormal findings of immunological markers were increased levels of cytokines, including interleukin (IL)-6 (eight, 88.9%), IL-4 (three, 37.5%), tumor necrosis factor (TNF)-α (one, 12.5%), IL-2 (one, 12.5%), and IL-10 (two, 25%). The details are summarized in Table 2.

**Table 2:** Laboratory findings of breast cancer patients with COVID-19.

**Variables**	**Value**
**Blood routine (unit; normal range)**	
White blood cell count (×10^9^/L; 3.5–9.5)	5.59 (4.66–6.42)
Decreased	2 (10.5%)
Increased	2 (10.5%)
Monocyte count (×10^9^/L; 0.1–0.6)	0.36 (0.31–0.48)
Decreased	1 (5.3%)
Increased	3 (15.8%)
Lymphocyte count (×10^9^/L; 1.1–3.2)	1.06 (0.85–1.51)
Decreased	10 (52.6%)
Neutrophil count (×10^9^/L; 1.8–6.3)	3.90 (2.70–4.37)
Decreased	2 (10.5%)
Increased	3 (15.8%)
Platelet count (×10^9^/L; 125–350)	196 (163–251)
Decreased	1 (5.3%)
Increased	2 (10.5%)
**Blood biochemistry (unit; normal range)**	
ALT (U/L; 5–35)	24 (15–47)
Increased	5 (26.3%)
AST (U/L; 8–40)	29 (19–37)
Increased	3 (15.8%)
CK (U/L; 26–140)	67 (48–106)
Increased	2 (10.5%)
LDH (U/L; 109–245)	209 (163–296)
Increased	6 (31.6%)
CKMB (ng/ml; 0–6.6)	0.5 (0.3–1.0)
TNI (ng/L; <26.2)	3.35 (1.95–5.025)
BUN (mmol/L; 2.5–6.1)	3.79 (3.215–6.865)
Increased	1 (16.7)
Creatinine (umol/L; 46–92)	63 (52–77)
Increased	3 (15.8%)
**Infection biomarkers (unit; normal range)**	
Procalcitonin (μg/L; <0.5)	0.09 (0.02–0.54)
Increased	2 (28.6%)
CRP (mg/L; 0–8)	10.84 (0.89–68.64)
Increased	5 (50%)
**Immunological markers (unit; normal range)**	
CD4/CD8 ratio (0.41–2.72)	1.61 (0.935–3.67)
Increased	3 (33.3%)
CD3 + T cell (%; 58.17–84.22)	68.27 (58.535–81.56)
Decreased	2 (22.2%)
Increased	2 (22.2%)
CD4 + T cell (%; 25.34–51.37)	41.32 (35.965–52.045)
Decreased	1 (11.1%)
Increased	2 (22.2%)
CD8 + T cell (%; 14.23–38.95)	34.64 (13.385–39.095)
Decreased	2 (22.2%)
Increased	2 (22.2%)
IL-6 (pg/ml; 0.10–2.90)	12.41 (5.7–29.45)
Increased	8 (88.9%)
IL-4 (pg/ml; 0.10–3.20)	1.925 (1.383–4.703)
Increased	3 (37.5%)
TNF α (pg/ml; 0.10–23.00)	3.25 (2.26–5.365)
Increased	1 (12.5%)
IL-2 (pg/ml; 0.10–4.10)	2.58 (2.33–3.70)
Increased	1 (12.5%)
IL-10 (pg/ml; 0.10–5.00)	3.48 (2.53–4.955)
Increased	2 (25%)

*PLT, platelet; ALT, alanine aminotransferase; AST, aspartate aminotransferase; CK, creatine kinase; LDH, lactate dehydrogenase; CKMB, creatine kinase-MB; TNI, troponin; CRP, C-reactive protein; IL, interleukin; TNF, tumor necrosis factor*.

**Figure 1:**
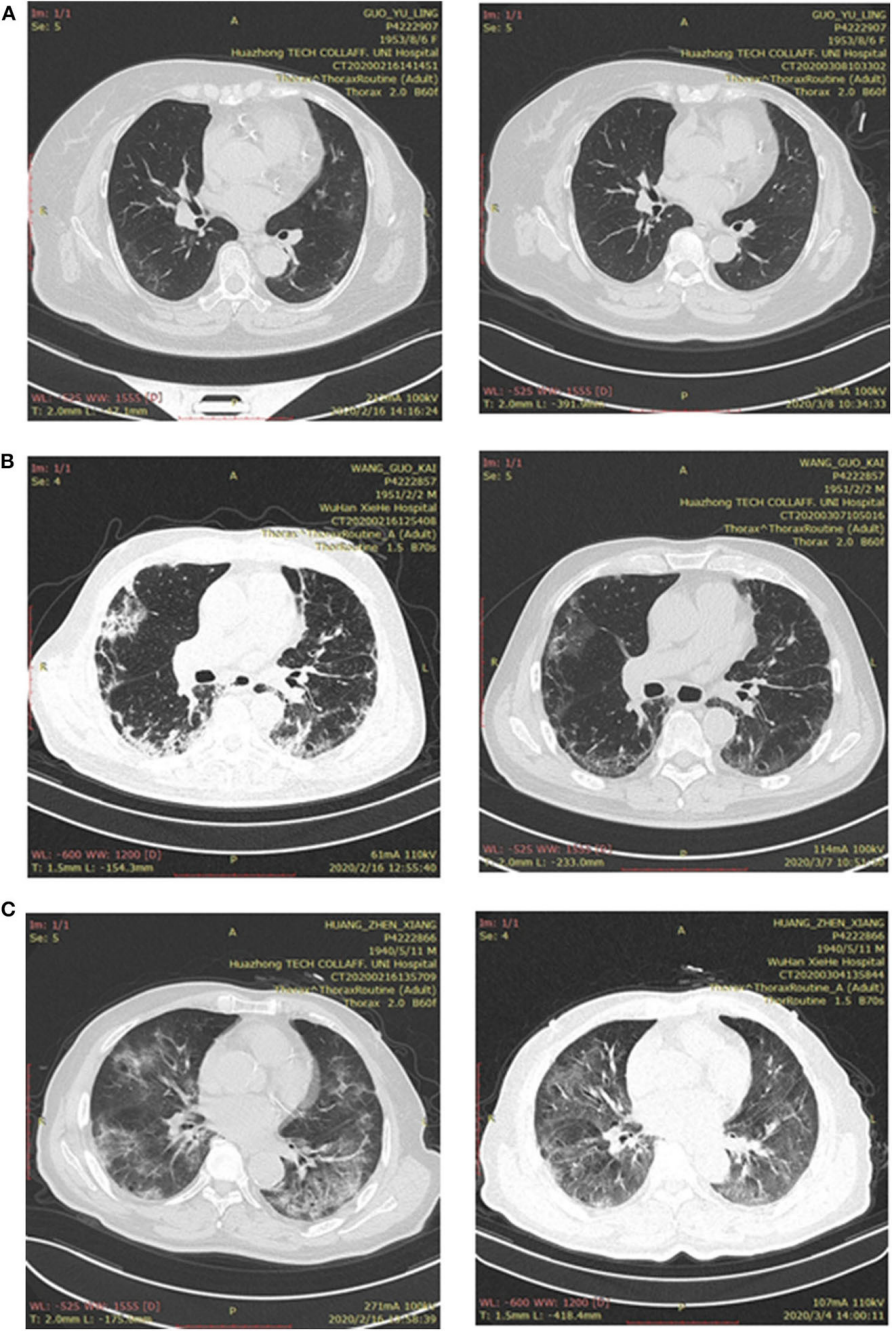
**(A)** Bilateral pneumonia in a patient with breast cancer. (Left side) Chest CT, showing bilateral ground-glass opacity, was performed on admission; (right side) CT was performed after treatment for COVID-19. **(B)** Bilateral pneumonia in a patient without cancer. (Left side) Chest CT, showing bilateral patchy shadowing, was performed on admission; (right side) CT was performed after treatment for COVID-19. **(C)** Bilateral pneumonia in a patient with other types of cancer. (Left side) Chest CT, showing bilateral ground-glass opacity, was performed on admission; (right side) CT was performed after treatment for COVID-19.

Of these breast cancer patients, only 15 patients received chest CT scan, and the radiologic features are shown in Table 3. Bilateral ground-glass opacity, the predominant CT imaging pattern, was observed in five patients (33.3%). Local patchy shadowing was observed in two (13.3%). Other abnormal features included local ground-glass opacity (one, 6.7%), bilateral patchy shadowing (one, 6.7%), and interstitial abnormalities (one, 6.7%). Cases of typical CT features are shown in Figure 1.

**Table 3:** Changes on the computed tomography of breast cancer patients with COVID-19.

**Characteristics**	**Value**
Bilateral ground-glass opacity	5/15 (33.3%)
Local ground-glass opacity	2/15 (13.3%)
Bilateral patchy shadowing	1/15 (6.7%)
Local patchy shadowing	1/15 (6.7%)
Interstitial abnormalities	1/15 (6.7%)

### Treatment and Clinical Outcomes

A small proportion of patients received treatment for SARS-COV-2 infection, including antiviral therapy (11, 31.4%), antibiotic therapy (nine, 25.7%), glucocorticoid therapy (three, 8.6%), immunomodulatory drug (six, 17.1%), and traditional Chinese medicine (eight, 22.9%). Some patients were just given routine anti-cancer treatment, including chemotherapy (five, 14.3%), radiotherapy (two, 5.7%), and targeted therapy (two, 5.7%). Only nine patients (25.7%) received oxygen inhalation through nasal cannula. None of the patients required invasive mechanical ventilation or intensive care unit (ICU) admission. Complications of myocardial injury, liver dysfunction, and renal dysfunction occurred during hospitalization in five cases (14.3%), two cases (5.7%), and two cases (5.7%), respectively. Up to the end of follow-up date, all the 35 patients were alive. The details are summarized in Table 4.

**Table 4:** Treatments and outcomes of breast cancer patients with COVID-19.

**Variables**	**Value**
**Treatments**	
Anti-tumor treatment	
Chemotherapy	5 (14.3%)
Radiotherapy	2 (5.7%)
Targeted therapy	2 (5.7%)
Antiviral therapy	11 (31.4%)
Antibiotic therapy	9 (25.7%)
Glucocorticoid	3 (8.6%)
Immunomodulatory drug	6 (17.1%)
Traditional Chinese medicine	8 (22.9%)
**Oxygen support**	
Nasal cannula	9 (25.7%)
Invasive mechanical ventilation	0
**Complications**	
Liver dysfunction	2 (5.7%)
Myocardial injury	5 (14.3%)
Renal dysfunction	2 (5.7%)
**Clinical outcomes**	
Alive	35 (100%)
Death	0

### Comparisons of Clinical Characteristics and Outcomes

Differences between COVID-19 patients with breast cancer and without cancer are shown in Table 5. Compared with non-cancer patients, breast cancer patients had a similar age [median age, 56 years (IQR, 42–62) vs. 57 years (IQR, 49–64)] and coexisting conditions, including hypertension [six (17.1%) vs. 17 (30.9%)], diabetes [four (11.4%) vs. 10 (18.2%)], and cardiovascular disease [two (5.7%) vs. seven (12.7%)]. At the onset of COVID-19, the breast cancer patients were more likely to be cases of asymptomatic infection [24 (68.6%) vs. 0; *P* < 0.001]. On admission, 31.4% of breast cancer patients were diagnosed as severe/critical, close to 20% of non-cancer patients (*P* = 0.314). Complications occurred in both breast cancer patients and non-cancer patients, including liver dysfunction (5.7% for breast cancer patients and 12.7% for non-cancer patients), myocardial injury (14.3% for breast cancer patients and 27.3% for non-cancer patients), and renal dysfunction (5.7% for breast cancer patients and 5.5% for non-cancer patients). During hospitalization, 4.3% of breast cancer patients needed oxygen inhalation compared to 69.1% of non-cancer patients (*P* < 0.001). Up to the final follow-up date, all the breast cancer patients and non-cancer patients were discharged and alive.

**Table 5:** Comparison of clinical characteristics and outcomes between breast cancer patients with COVID-19 and non-cancer patients with COVID-19.

	**Breast cancer patients with COVID-19 (*N* = 35)**	**Non-cancer patients with COVID-19 (*N* = 55)**	***P*-value**
Age, median (IQR)—year	56 (42–62)	57 (49–64)	0.73
**Sex**			
Male	0	23 (41.8%)	<0.001
Female	35 (100%)	32 (58.2%)	
**Comorbidities**			
Number of comorbidities per patient >2	3 (8.6%)	8 (14.5%)	0.518
Hypertension	6 (17.1%)	17 (30.9%)	0.215
Diabetes	4 (11.4%)	10 (18.2%)	0.553
Cardiovascular disease	2 (5.7%)	7 (12.7%)	0.473
Chronic lung disease	0	0	
Common pulmonary infection	0	0	
Anemia	3 (8.6%)	0	0.056
Asymptomatic	24 (68.6%)	0	<0.001
Clinical manifestations of COVID-19	11 (31.4%)	55 (100%)	
Fever	6/11 (54.5%)	40/55 (72.7%)	0.287
Cough	8/11 (72.7%)	33/55 (60%)	0.513
Shortness of breath	2/11 (18.2%)	20/55 (36.4%)	0.312
Chest tightness	4/11 (36.4%)	22/55 (40%)	>0.99
Dyspnea	2/11 (18.2%)	8/55 (14.5%)	>0.99
Anorexia	1/11 (9.1%)	16/55 (29.1%)	0.264
Myalgia	3/11 (27.3%)	14/55 (25.5%)	>0.99
Sore throat	0	9/55 (16.4%)	0.337
Diarrhea	3/11 (27.3%)	10/55 (18.2%)	0.678
Digestive symptoms	3/11 (27.3%)	13/55 (23.6%)	
**Disease severity**			
Mild	24 (68.6%)	44 (80%)	0.314
Severe/critical	11 (31.4%)	11 (20%)	
**Complications**			
Liver dysfunction	2 (5.7%)	7 (12.7%)	0.473
Myocardial injury	5 (14.3%)	15 (27.3%)	0.196
Renal dysfunction	2 (5.7%)	3 (5.5%)	>0.99
MODS	0	3 (5.5%)	0.279
**Treatments**			
Oxygen inhalation	9 (25.7%)	38 (69.1%)	<0.001
Invasive mechanical ventilation	0	0	
ECMO	0	0	
ICU admission	0	0	
**Clinical outcomes**			
Alive	35 (100%)	55 (100%)	>0.99
Died	0	0	

*IQR, interquartile range; MODS, multiple organ dysfunction; ECMO, extracorporeal membrane oxygenation; ICU, intensive care unit*.

Differences between COVID-19 patients with breast cancer and other types of cancer are shown in Table 6. The median age of other types of cancer patients was 58 years (IQR, 49–62), matched to the breast cancer patients. Among other types of cancer patients with COVID-19, 38 patients (46.9%) were male, 26 (32.1%) were cases of asymptomatic infection, and lung cancer (12, 14.8%) was the most common type of cancer. Compared with breast cancer patients, other types of cancer patients were more likely to develop a severe/critical disease [51 (63%) vs. 11 (31.4%); *P* = 0.002]. Common complications among other types of cancer patients included liver dysfunction (seven, 8.6%), myocardial injury (21, 25.9%), renal dysfunction (14, 17.3%), and multiple organ dysfunction (nine, 11.1%). During hospitalization, more other types of cancer patients dramatically required oxygen inhalation [47 (58%) vs. nine (25.7%); *P* = 0.002] than breast cancer patients. Up to the end of the follow-up date, the mortality rate reached 9.9% among other types of cancer patients, close to the zero mortality of breast cancer patients.

**Table 6:** Comparison of clinical characteristics between breast cancer patients with COVID-19 and other types of cancer patients with COVID-19.

	**Breast cancer patients with COVID-19 (*N* = 35)**	**Other types of cancer patients with COVID-19 (*N* = 81)**	***P*-value**
Age, median (interquartile range)—year	56 (42–62)	58 (49–62)	0.416
**Sex**			
Male	0	38 (46.9%)	<0.001
Female	35 (100%)	43 (53.1%)	
**Type of cancer**			
Breast cancer	35 (100%)	NA	
Lung cancer	NA	12 (14.8%)	
Cervical cancer	NA	7 (8.6%)	
Thyroid cancer	NA	7 (8.6%)	
Leukemia	NA	6 (7.4%)	
Colon cancer	NA	6 (7.4%)	
Gastric cancer	NA	6 (7.4%)	
Myeloma	NA	6 (7.4%)	
Pancreatic cancer	NA	5 (6.2%)	
Liver cancer	NA	4 (4.9%)	
Lymphoma	NA	4 (4.9%)	
Rectum cancer	NA	3 (3.7%)	
Bladder cancer	NA	3 (3.7%)	
Ovarian cancer	NA	3 (3.7%)	
Nasopharynx cancer	NA	2 (2.5%)	
Myelodysplastic syndrome	NA	2 (2.5%)	
Esophageal cancer	NA	1 (1.2%)	
Osteosarcoma	NA	1 (1.2%)	
Liposarcoma	NA	1 (1.2%)	
Meningioma	NA	1 (1.2%)	
**Comorbidities**			
Number of comorbidities per patient >2	3 (8.6%)	20 (24.7%)	0.073
Hypertension	6 (17.1%)	18 (22.2%)	0.624
Diabetes	4 (11.4%)	12 (14.8%)	0.773
Cardiovascular disease	2 (5.7%)	7 (8.6%)	0.721
Chronic lung disease	0	6 (7.4%)	0.109
Common pulmonary infection	0	6 (7.4%)	0.109
Anemia	3 (8.6%)	6 (7.4%)	>0.99
Asymptomatic	24 (68.6%)	26 (32.1%)	<0.001
Clinical manifestations of COVID-19	11 (31.4%)	55 (67.9%)	
Fever	6/11 (54.5%)	34/55 (61.8%)	0.741
Cough	8/11 (72.7%)	32/55 (58.2%)	0.505
Shortness of breath	2/11 (18.2%)	12/55 (21.8%)	>0.99
Chest tightness	4/11 (36.4%)	12/55 (21.8%)	0.44
Dyspnea	2/11 (18.2%)	12/55 (21.8%)	>0.99
Anorexia	1/11 (9.1%)	9/55 (13.4%)	0.683
Myalgia	3/11 (27.3%)	10/55 (18.2%)	0.678
Sore throat	0	3/55 (5.5%)	>0.99
Diarrhea	3/11 (27.3%)	10/55 (18.2%)	0.678
Digestive symptoms	3/11 (27.3%)	16/55 (29.1%)	>0.99
**Disease severity**			
Mild	24 (68.6%)	30 (37%)	0.002
Severe/critical	11 (31.4%)	51 (63%)	
**Complications**			
Liver dysfunction	2 (5.7%)	7 (8.6%)	0.721
Myocardial injury	5 (14.3%)	21 (25.9%)	0.227
Renal dysfunction	2 (5.7%)	14 (17.3%)	0.143
MODS	0	9 (11.1%)	0.056
Antiviral therapy	11/11 (100%)	50/55 (90.9%)	0.58
Antibiotic therapy	9/11 (81.8%)	46/55 (83.6%)	>0.99
Immunomodulatory drug	6/11 (54.5%)	37/55 (67.3%)	0.495
Glucocorticoid	3/11 (27.3%)	13/55 (23.6%)	>0.99
Traditional Chinese medicine	8/11 (72.7%)	24/55 (43.6%)	0.104
**Treatments**			
Oxygen inhalation	9 (25.7%)	47 (58%)	0.002
Invasive mechanical ventilation	0	5 (6.2%)	0.188
ECMO	0	0	
ICU admission	0	8 (9.9%)	0.103
**Clinical outcomes**			
Alive	35 (100%)	73 (90.1%)	0.103
Died	0	8 (9.9%)	

### The Effect of the Clinical Characteristics of COVID-19-Infected Breast Cancer Patients on the Severity of COVID-19

Among breast cancer patients, we analyzed the effect of age, comorbidities, abnormal chest CT findings, and chemotherapy, radiotherapy, and targeted therapy within 1 month of COVID-19 diagnosis on the severity of COVID-19. The univariate analysis showed that age, comorbidities, and abnormal chest CT findings were related to disease severity and may be factors that affect the development of the disease (Table 7). Furthermore, a multivariate analysis (Table 8) indicated that only age (OR, 1.325; 95% CI, 1.075–1.634; *P* = 0.008) could be an independent factor affecting the severity of COVID-19 in breast cancer patients.

**Table 7:** Univariate analysis of the correlation between clinical factors and severity of disease in breast cancer patients with COVID-19.

	**Breast cancer patients with COVID-19 (*****N*** **=** **35)**		***P*-value**
	**Mild (*N* = 24)**	**Severe/critical (*N* = 11)**	
Age, median (interquartile range), year	45.5 (40.3–57.5)	67 (60–71)	<0.001
Fever	0	6 (54.5%)	
Cough	0	8 (72.7%)	
Chest tightness	0	4 (36.4%)	
Diarrhea	0	3 (27.3%)	
Shortness of breath	0	2 (18.2%)	
Dyspnea	0	2 (18.2%)	
Anorexia	0	1 (9.1%)	
Muscle ache	0	3 (27.3%)	
Asymptomatic	24 (100%)	0	
Comorbidities	4 (16.7%)	8 (72.7%)	0.002
Number of comorbidities per patient >2	1 (4.2%)	2 (18.2)	0.227
Hypertension	2 (8.3%)	4 (36.4%)	0.063
Diabetes	1 (4.2%)	3 (27.3%)	0.082
Cardiovascular disease	1 (4.2%)	1 (9.1%)	>0.99
Anemia	2 (8.3%)	1 (9.1%)	>0.99
Abnormal chest CT findings	0/4	10/11 (90.9%)	0.004
Chemotherapy within 1 month	5 (20.8%)	1 (9.1%)	0.640
Radiotherapy within 1 month	2 (8.3%)	0	0.556
Targeted therapy within 1 month	2 (8.3%)	0	0.556

**Table 8:** Multivariate analysis for the risk of disease severity in breast cancer patients with COVID-19.

**Clinical factor**	**Regression coefficients**	**OR**	**95% CI**	***P*-value**
Age	0.281	1.325	1.075–1.634	0.008
With Comorbidities	−2.841	0.058	0.001–3.789	0.182

*OR, odds ratio; CI, confidence interval*.

## Discussion

In this retrospective study, we analyzed the clinical data of 35 breast cancer patients with COVID-19. Among these breast cancer patients, the median age of the breast cancer patients was 56 years (IQR, 42–62). Common comorbidities included hypertension (six, 17.1%), myelosuppression (four, 11.4%), diabetes (four, 11.4%), anemia (three, 8.6%), and cardiovascular disease (two, 5.7%). At the onset of illness, most patients (24, 68.6%) were asymptomatic infections and diagnosed as mild, and the remaining 11 patients (31.4%) with clinical symptoms were severe/critical. Common symptoms among the 11 patients included fever, cough, fatigue, and chest tightness. Our results showed elevated levels of ALT, AST, CK, LDH, IL-6, IL-4, IL-10, and CRP and a decreased level of lymphocyte count in a proportion of patients. Bilateral and local ground-glass opacity was a typical hallmark of CT scan for the breast cancer patients. Major complications during hospitalization included liver dysfunction, myocardial injury, and renal dysfunction. At the final follow-up date, all patients were discharged and alive.

Previous studies reported that cancer patients carry higher severity of COVID-19 and poorer prognosis compared with non-cancer patients infected with COVID-19 (11). However, due to the small sample size and insufficient clinical data, more evidence is needed to verify this conclusion (8, 9). Therefore, in our study, the clinical characteristics and the outcomes of 55 COVID-19 patients without cancer and 81 COVID-19 patients with other types of cancer as controls were compared with those of the 35 breast cancer patients, respectively. COVID-19 patients with breast cancer had similar age and underlying comorbidities with common COVID-19 patients. Among non-cancer patients, there was no asymptomatic infection, which was substantially different with breast cancer patients. Interestingly, we found that there were no significant differences in disease severity and mortality rate between breast cancer patients and non-cancer patients. In contrast to previous studies, COVID-19 patients with breast cancer were not more severe, and their prognosis was not worse than those of common COVID-19 patients. To our knowledge, presymptomatic or asymptomatic transmission is responsible for around 50% of the overall attack rate in COVID-19 outbreaks (5, 12), which should be seriously considered by clinicians.

At the onset of COVID-19, 32.1% of other types of cancer patients were in asymptomatic state, substantially smaller than breast cancer patients. Other types of cancer patients were more likely to develop severe/critical condition, which indicated that COVID-19 patients with other types of cancer patients were more severe than patients with breast cancer. Complications of liver dysfunction, myocardial injury, and renal dysfunction may occur in both breast cancer patients and other types of cancer patients. During hospitalization, most other types of cancer patients received oxygen inhalation; five (6.2%) and eight (9.9%) required invasive mechanical ventilation and ICU admission, respectively. The mortality rate of other types of cancer patients was 9.9%, which was not significantly higher than breast cancer patients. Among the other types of cancer patients, the most common cancer type was lung cancer (12, 14.8%). Lung cancer patients, with worse baseline pulmonary function, progress more rapidly and are more likely to develop severe events with SARS-COV-2 infection (7). The morbidity and the mortality of COVID-19 are closely related to elderly age and underlying diseases (13). Our data showed that there were no differences in age and comorbidities between COVID-19 patients with breast cancer and other types of cancer, which may account for the similar prognosis of the two groups. In addition, our results, through univariate and multivariate analyses, indicated that elderly breast cancer patients with COVID-19 were more likely to develop a severe illness.

However, there were several limitations in our study. Firstly, this study was retrospective and was based on a relatively small size of samples. Secondly, clinical information was insufficient, such as tumor stage, laboratory parameters, and chest CT features, such that we could not investigate the risk factors contributing to severe events in COVID-19-infected breast cancer patients. Finally, the sex ratio of breast cancer patients was significantly different from that of the control groups, which may influence the conclusion of our study.

## Data Availability Statement

All datasets generated for this study are included in the article/supplementary material.

## Ethics Statement

The studies involving human participants were reviewed and approved by Ethics Committee of the Tongji Medical College of Huazhong University of Science and Technology. Written informed consent for participation was not required for this study in accordance with the national legislation and the institutional requirements.

## Author Contributions

BZ and RX: conceptualization and writing—original draft preparation. SH: methodology and software. YY, YZ, XL, and WD: resources and data curation. JC: writing—reviewing and editing. YL: visualization and supervision. All authors contributed to the article and approved the submitted version.

## Conflict of Interest

The authors declare that the research was conducted in the absence of any commercial or financial relationships that could be construed as a potential conflict of interest.
